# Expression of interleukine-8 as an independent prognostic factor for sporadic colon cancer dissemination

**Published:** 2014-06-25

**Authors:** A Nastase, L Paslaru, V Herlea, M Ionescu, D Tomescu, N Bacalbasa, S Dima, I Popescu

**Affiliations:** * Fundeni Clinical Institute, Bucharest, Romania *The two authors had an equal contribution in the article

**Keywords:** interleukine-8, biomarkers, colon cancer, metastasis, qPCR

## Abstract

Abstract

Aim: The aim of our study was to investigate the gene and serum protein expression profiles of IL-8 in colon cancer and associated hepatic metastasis and to correlate these results with clinicopathologic variables of the patients.

Materials and methods: IL-8 was evaluated by qPCR and ELISA in a total number of 62 colon cancer patients (n=42 by qPCR and n=20 by ELISA) in normal and tumoral tissue specimens and serum samples respectively. Additionally synchronous metastasis from 5 of these patients were also collected at the time of surgery and analyzed by qPCR.

Results: IL-8 was up regulated in all analyzed tumoral samples compared with normal tissue (P-value = 0.01) and higher expressed in metastatic tissues compared with tumoral tissues (P -value= 0.03). The median expression of IL-8 in patients over 60 years old was found to be higher compared with the median expression of IL8 in patients less than 60 years old (3.89 compared with 14.69, P -value= 0.005). According to tumor grading, we found that IL-8 in tumors with well differentiated adenocarcinoma have a median mRNA expression of 9.78 compared with a median mRNA IL8 expression of 26.63 in moderate or poor differentiated adenocarcinoma.

Levels of IL-8 determined in serum were statistically significant correlated with preoperative carcinoembryonic antigen level (P -value= 0.003, R=0.57) and with distant metastasis (P-value =0.008). Serum level of IL-8 increased proportionally along with TNM tumor stage and was found to be statistically significant correlated with C-reactive protein (P -value, R=0.64). Colon cancer patients had higher IL-8 levels as determined by ELISA (median value= 29.64 pg/ml) compared with healthy controls (median value= 4.86 pg/ml).

Discussions: Our results provide additional support for the role of inflammation in colon cancer and indicate that IL-8 could be further validated in association with other already used markers for prognostic and diagnostic of evolutional disease in colon cancer patients.

Brief abstract

By investigating the gene and serum protein expression profiles of IL-8 in colon cancer and associated hepatic metastasis, we found correlations between these results and clinicopathological variables of the patients. IL-8 is involved in colon cancer progression and could be monitored in a panel with other biomarkers as an early indicator of the tumor’s evolution.

## Introduction

It is well established that there is a profound link between colon cancer and chronic inflammation [**1,2**]. Infectious agents lead to chronic inflammation at the site of tumor development [**3**], which attributes to the inflammation of a causative part in [**4**] the initiation, promotion and progression of cancer. 

 IL-8 (CXCL8) is involved in a variety of physiopathological processes [**5**] and along with other members of TNF superfamily were shown to be involved in the proliferation, invasion and metastasis of several cancer [6,7] including colon cancer [**8,10**]. 

 IL-8 is a member of neutrophil-specific CXC-chemokines family with ELR (Glu-Leu-Arg) motif [**11,12**] that belong to G-protein-coupled receptor family [**13**] and exert its function through biding to two receptors, CXCR1 and CXCR2. Its activation is mediated through NF-kB pathway [**14**]. 

 At the tumoral level, IL-8 influences the tumor growth, survival, invasion, angiogenesis, metastasis [**15**], resistance and recurrence [**8**] and its overexpression is associated with a poor prognosis [**16**]. 

 The aim of our paper was to investigate the potential links between gene and protein IL-8 expression with clinicopathologic variables of colon cancer patients and to evaluate its role in colon cancer progression.

## Materials and methods 

Patient selection and classification The ethics committee of Fundeni Clinical Institute approved our study according to the in force legislation. All the patients signed a written informed consent. A total number of 62 patients with sporadic colon cancer were used in our study (n=42 for transcriptomic study and n=20 for ELISA tests). Additionally, blood was drawn from 20 healthy subjects who represented the control group for ELISA tests. All the patients had no records of oncological treatment prior to surgery. Clinicopathologic data are shown in Table 1.

**Table 1:** Clinicopathologic features of the patients

Parameter	N ( %)
Age, years (average ± SD)	63.94 ± 8.5
Men	39 (62.9)
Women	23 (37.1)
TNM tumor stage	
I	3 (4.8 )
II	20 (32.3)
III	20 ( 32.3)
IV	19 (30.6 )
Lymph node status	
0	26 (42.0)
1	17 (27.4)
2	19 (30.6 )
pT	
2	5 (8.1)
3	56 (90.3)
4	1 (1.6)
Distant metastasis	
M0	43 (69.4)
M1	19 (30.6 )
Differentiation degree	
G1	47 (75.8 )
G1-G2	7 (11.3)
G2	6 (9.7)
G3	2 (3.2)

Tissue and serum sample collection 

 Blood samples were collected preoperatively for each patient. Blood was drawn under fasting conditions in vacutainers without anticoagulant and serum was collected after centrifugation and stored at -80°C. 

 During each patient’s surgery, normal and tumoral samples were collected. In the case of 5 patients, specimens from synchronous metastasis were also collected. A trained pathologist made a histological diagnostic from samples immersed in formalin and then paraffin embedded. The transcriptomic study was performed by using tissue samples that were immediately snapped frozen in liquid nitrogen. 

 RNA extractions 

 Total RNA was isolated from normal, tumoral and when the case from metastatic samples by means of tri reagent (Sigma, St. Louis, MO) according to the manufacturer`s instructions. Purification was done with RNeasy Mini Kit (QIAGEN, Valencia, CA). The quantity and quality of the total RNA were assessed by spectrophotometry (Nano Drop 1000; Thermo Scientific, Arlington, TX) and by lab-on-a-chip Agilent 2100 technology (Agilent Technology, Santa Clara, CA). All samples had a 260:280 nm ratio greater than 1.8, a 28S:18S ratio greater than 1.5, and an RNA integrity number greater than 7. 

 Real-time qPCR 

 cDNA was obtained from 2 µg of total RNA, by using High Capacity cDNA Archive Kit (ABI, Foster City, CA) in a total volume of 20 µL. Samples were diluted to 2 ng/µL, and qPCR amplification was performed in triplicate for each sample in a total volume of 25 µL under the following conditions: 95°C for 10 minutes, 95°C for 15 seconds, and 1 minute at 60°C for 40 cycles. 

 The 2-step relative quantification was performed with the 7300 Real-Time PCR (ABI, Foster City, CA) system with hydrolysis probes labeled with 6-carboxyfluorescein. Normal tissues were used as control and mRNA was normalized to reference genes RPLP0 (20X). Data were analyzed with SDS 1.4 software by using the comparative Ct method [2^(-delta delta Ct)]. The tested gene was IL-8 (Hs00174103_m1). 

 CEA testing 

 Carcinoembryonic antigen (CEA) was assessed for each patient by chemiluminescence in preoperative serum samples by using a commercial kit (Cobas Core, Roche Diagnostic Systems). 

 Enzyme-linked immunosorbent assay 

 Serum concentration of IL-8 was determined by using a sandwich ELISA system (Uscn Life Science, Wuhan, China) according to the manufacturer’s instructions. The color change was detected spectrophotometrically at a wavelength of 450 nm and the concentrations of the samples were interpolated on the standard curve. 

 C-reactive protein (CRP) 

 The C-reactive protein (CRP) levels were measured by means of a commercially available assay by using Abbott c8000 automated equipment according to the manufacturer protocol (Abbott, North Chicago, IL). 

 Statistical analysis 

 Calculations were performed with Graph Pad Prism 5 (Graph Pad Software Inc, San Diego, CA). Data was tested for normal distribution by using the Kolmogorov-Smirnov and Shapiro-Wilk tests. Comparisons among the groups were made by means of the Kruskal-Wallis and Mann Whitney U tests. A P-value less than or equal to 0.05 was considered statistically significant. 

## Results

Expression of mRNA IL-8 in colon cancer tissues

**Fig. 1:**
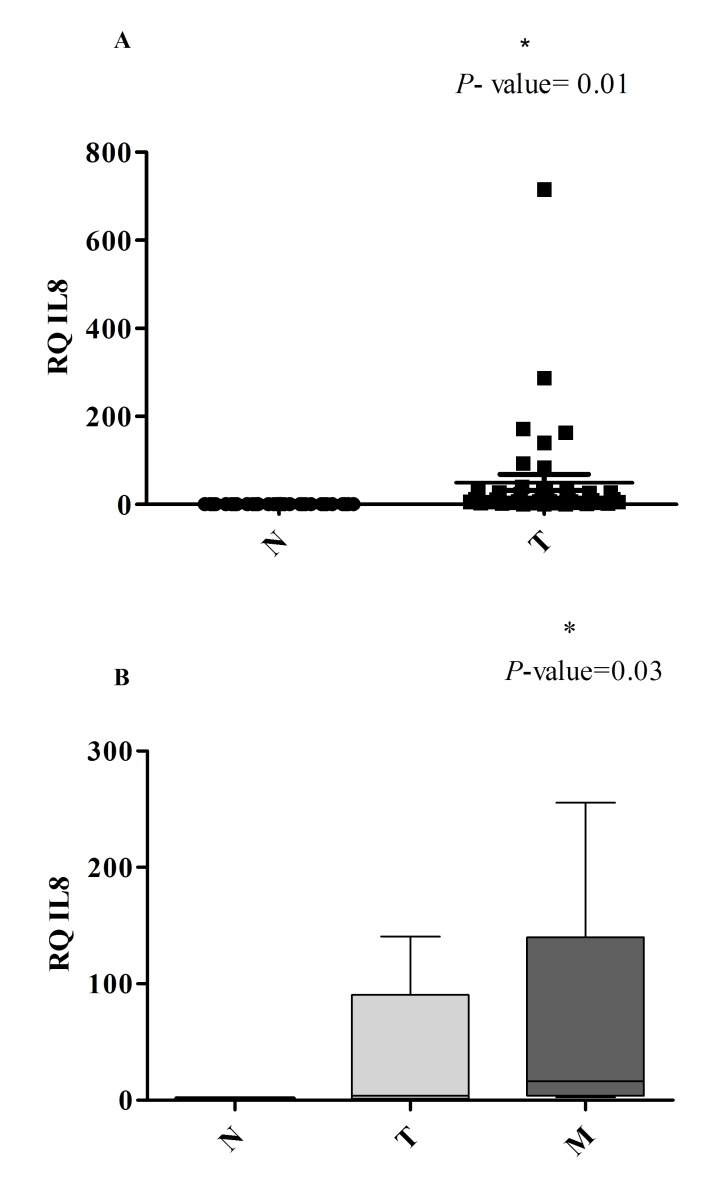
qPCR measurements of IL-8 gene expression in (A) tumoral tissue compared with normal tissue and (B) tumoral and metastatic tissue compared with normal tissue. The values are expressed as means of three independent replicates

 mRNA IL-8 gene expression was found to be up-regulated in tumoral tissue compared with normal tissues (P -value= 0.01). In patients in whom the metastatic tissue was available, IL-8 mRNA was found to be higher expressed compared with the tumoral tissue (P -value= 0.03) (**Fig. 1**).

**Fig. 2:**
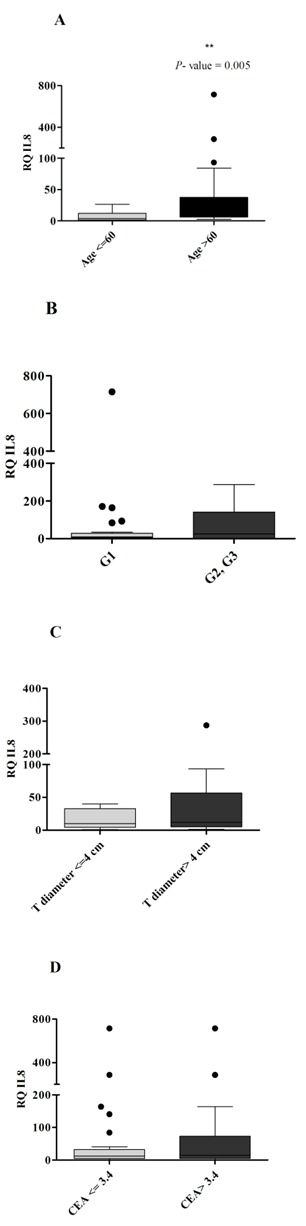
Differential IL-8 gene expression in (A) patients below and above 60 years old (B) well differentiated and moderate and poor differentiated tumors (C) tumors with the diameter less than or greater than 4 cm (D) patients with CEA below or greater than 3.4 ng/ml

 The analysis of IL-8 gene expression in correlation with the clinicopathologic features of the patients revealed a differential expression in different patients groups (**Fig. 2**).

 Association between IL-8 assessed by ELISA and clinicopathologic parameters of the patients

 Enzyme linked immunosorbent assay (ELISA) on the serum samples detected measurable levels in 90% of the tested samples for IL-8.

 The levels of IL-8 determined by ELISA in the serum of the patients were found to be statistically correlated with the level of the carcinoembryonic antigen (CEA) determined in the preoperative serum of the patients (P-value =0.003, R= 0.64), with tumor stage (P -value =0.01, R= 0.57), with distant metastasis (P-value =0.008) and with C-reactive protein (P -value =0.003, R= 0.64).

**Fig. 3:**
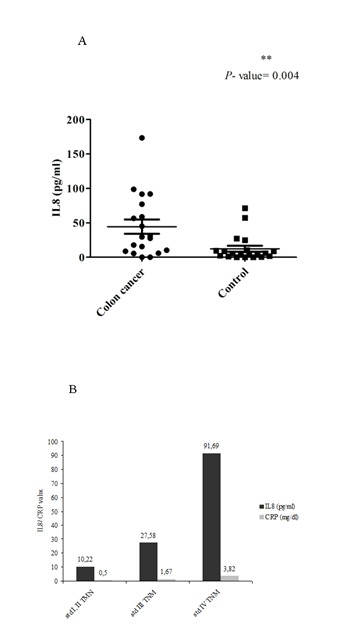
(A) IL-8 assessed by ELISA in colon cancer patients versus control subjects and (B) IL-8 assessed by ELISA and CRP in colon cancer patients with tumors stage I and II, III and IV TNM

 IL-8 concentration was found to be statistically higher (P -value =0.004) in colon cancer patients (median value= 29.64 pg/ml) compared with the concentration of IL-8 determined in a group of 20 healthy controls (median value= 4.86 pg/ml) (Fig. 3A). The area under the curve for IL-8 was determined to be 0.765 (95%CI: 0.609-0.922).

The level of IL-8 determined by ELISA was found to increase from tumors with stage I and II TNM to stage III TNM and to stage IV TNM (P -value= 0.027). The same tendency was observed for C-reactive protein (Fig. 3 B).

 Assessing the serum level of IL-8 depending on lymph node stage (number of negative/ positive lymph nodes) we found out that in patients without affected lymph nodes, the level of IL-8 is 14.0 pg/ml while in patients with one or more than one affected lymph nodes the level of IL-8 increases to 56.52 pg/ml. 

## Discussions

The goal of our study was to evaluate the involvement of IL-8 in colon cancer progression by assessing its gene and protein expression with clinicopathologic features of the patients. 

 IL-8 is produced by both normal as well as tumor cells and is implicated in the initiation and amplification of inflammatory processes that occur in cancer [**17,18**]. 

 In normal cells, IL-8 is secreted at very low levels [**14**] but its production is stimulated by other cytokines [**19,21**], bacteria [**22,23**] or stress [**24,26**]. 

 IL-8 favors both metastatic spread of cancer cells as well as angiogenesis and tumor growth [**27,16**]. 

 In our study, we showed that IL-8 is unregulated in all analyzed tissues and its mRNA level increases from normal to tumoral and further to metastatic tissue. Further different gene expression level for IL-8 was obtained according to tumor grading (well differentiated versus moderate and poorly differentiated adenocarcinoma). 

 Our data demonstrate a statistical correlation between IL-8 determined by ELISA and clinicopathologic features of the patients. 

 IL-8 measured by ELISA was able to significantly distinguish between case and control groups. 

 Moreover, a statistical correlation between IL-8 with CRP is suggestive for the inflammatory component of colon cancer. 

 The results obtained in our study demonstrated that IL-8 has a higher gene and protein expression in colon cancer samples and its level increases as the disease progresses and metastasizes. 

 IL-8 is an important cytokine that is involved in colon cancer progression and could be monitored in panel with other biomarkers as an early indicator of the tumor’s evolution. 
